# Ergonomics in Dentistry

**DOI:** 10.5005/jp-journals-10005-1229

**Published:** 2014-04-26

**Authors:** Anshul Gupta, Manohar Bhat, Tahir Mohammed, Nikita Bansal, Gaurav Gupta

**Affiliations:** Senior Resident, Department of Pedodontics, SP Medical College and Associated Group of hospitals, Bikaner, Rajasthan, India; Professor and Head, Department of Pedodontics, Jaipur Dental College, Jaipur Rajasthan, India; Assistant Professor, Department of Prosthodontics, Government Dental College Jaipur, Rajasthan, India; Lecturer, Department of Pedodontics, Jaipur Dental College, Jaipur Rajasthan, India; Senior Lecturer, Department of Pedodontics, Jaipur Dental College, Jaipur Rajasthan, India

**Keywords:** Ergonomics, Carpal tunnel syndrome, Musculo‑ skeletal disorders, Lower back pain

## Abstract

Ergonomics is much broader than preventing work‑related musculoskeletal disorders. The successful application of ergo‑ nomics assures high productivity, avoidance of illnesses and injuries, and increased satisfaction among workers. Unsuc‑ cessful application, on the other hand, can lead to work‑related musculoskeletal disorders (MSDs). This article sets forth broad important background information on ergonomics so that the dental practitioner can have a general awareness of ergonomic risk factors as well as some basis for understanding the ongo‑ ing dialogue about ergonomics, its diagnosis, treatment, and regulation. This article provides alternatives to be considered by the practitioner in light of the practitioner's own circumstances, experiences and goals. A practitioner wishing to improve his or her work environment, for whatever reason, may wish to follow an incremental approach to such efforts, as is briefy discussed here.

**How to cite this article: **Gupta A, Bhat M, Mohammed T, Bansal N, Gupta G. Ergonomics in Dentistry. Int J Clin Pediatr Dent 2014;7(1):30-34.

## INTRODUCTION

Ergonomics can be defined as *‘*an applied science concerned with designing and arranging things people use so that the people and things interact most Efficiently and safely’.

The term work-related musculoskeletal disorders (MSDs) refers to musculoskeletal disorders to which the work environment contributes significantly or to musculoskeletal disorders that are made worse or longer lasting by work conditions or workplace risk factors. Common examples of such workplace risk factors include jobs requiring repetitive, forceful or prolonged exertions of the hands; frequent or heavy lifting, pushing or pulling, or carrying of heavy objects and prolonged awkward postures. The level of risk depends on the intensity, frequency and duration of the exposure to these conditions.

### Reasons for Early Retirement among Dentists^1^

- Musculoskeletal disorders (29.5%)
- Cardiovascular disease (21.2%)
- Neurotic symptoms (16.5%)
- Tumors (7.6%)
- Diseases of the nervous system (6.1%).

Good working ergonomics is essential so that work capability, Efficiency and high clinical level of treatment can be maintained throughout the working life of dental professionals. The scope of ergonomics in dentistry is large: it ranges from chemistry between the dental team to lighting, noise and odor conditions and naturally to the used equipment and software. The treatment environment with the patient chair, dental unit, operating light, dynamic and hand instrumentation, cabinetry and peripheral equipment must be flexible. They need to adapt and guarantee good working postures, sufficient lighting and easy access to required instrumentation and materials for different working practices, clinical procedures and patient types.

## TYPES OF MSDs

Musculoskeletal disorders come in a variety of forms. This article includes general information about the primary types of MSDs that dentists have to face.

### Back Problems

Lower Back Pain

Between 70 and 90% of people have recurrent episodes of pain, and one-third of patients continue to have persistent, recurrent or intermittent pain after their first episode. In addi­tion to the difficulty with healing, the degenerative process is ongoing with age, and many patients do not minimize potential risk factors. All of this can contribute to continue episodes of low back pain (LBP).^2^

The cause of LBP is often multifactorial but combined motions of lumbar flexion with rotation increase risk to the lumbar disk. This is further exacerbated by inflexibilities around the hips and pelvis as well as relative weakness of the stabilizers of the lumbar spine, including the abdominal and gluteal muscles. Furthermore, back pain can exist due to abnormal postures, relative weakness and decreased endurance, and then exacerbated by a ‘specific’ injury.

Upper Back Pain

While not as common as lower back pain, some individuals report extensive pain in the mid and upper back. The thoracic spine is designed for support in standing and for caging the vital organs and is quite strong. It only rarely experiences symptoms of degeneration since there is little movement and great stability.

Probably, a more frequent cause of mid back pain is muscular pain from the postural muscles and scapular muscles. The contributions of abnormal posture, static postures, poor strength and endurance, and overall individual conditioning need to be taken into account.

### Hand and Wrist Problems

A predominant cause of repetitive motion hand disorders is constant flexion and extension motions of the wrist and fingers. Chronic, repetitive movements of the hand and wrist, especially with the hand in ‘pinch’ position, seem to be the most detrimental. Other common contributing fac­tors to hand and wrist injuries include movements in which the wrist is deviated from neutral posture into an abnormal or awkward position, working for too long period without allowing rest or alternation of hand and forearm muscles; mechanical stresses to digital nerves from sustained grasps to sharp edges on instrument handles, forceful work and extended use of vibratory instruments.

Some of the common hand and wrist conditions are as follows:

- Tendinitis/tenosynovitis
- DeQuervain's disease
- Trigger finger
- Carpal Tunnel syndrome^3^
- Guyon's syndrome.

## SIGNS OF MSDs

- Decreased range of motion
- Loss of normal sensation
- Decreased grip strength
- Loss of normal movement
- Loss of coordination.

## SYMPTOMS OF MSDs

- Excessive fatigue in the shoulders and neck
- Tingling, burning or other pain in arms
- Weak grip, cramping of hands
- Numbness in fingers and hands
- Clumsiness and dropping of objects
- Hypersensitivity in hands and fingers.

## DENTAL RISK FACTORS

Following are recognized as important risk factors for musculoskeletal disorders among dental professionals, especially when occurring at high levels and in combination.

### Awkward Postures

More stress is placed on the spinal disks when lifting, lowering, or handling objects with the back bent or twisted compared with when the back is straight. Manipulative or other tasks requiring repeated or sustained bending or twisting of the wrists, knees, hips, or shoulders also imposed increased stresses on these joints. Activities requiring frequent or prolonged work over shoulder height can be particularly stressful.

Dental personnel assume these awkward positions for the following reasons:

- To coordinate the relative positions between dentist and assistant.
- To obtain optimal view of teeth within the patient's mouth.
- To provide a comfortable position for the patient.
- To maneuver complex equipment and reach for instru­ments.

### Forceful Exertions

Tasks that require forceful exertions (like tooth extractions) place higher loads on the muscles, tendons, ligaments and joints. Prolonged experiences of this type can give rise to not only feelings of fatigue but may also lead to musculoskeletal problems when there is inadequate time for rest or recovery. Force requirements may increase with:

- Use of an awkward posture.
- The speeding up of movements.
- Use of small or narrow tool handles that lessen grip capacity.
- Increased slipperiness of the objects handled.
- Use of the index finger and thumb to forcefully grip an object (i.e. a pinch grip compared with gripping the object).

### Repetitive Motions

If motions are repeated frequently and for prolonged periods, fatigue and muscle-tendon strain can accumulate. Effects of repetitive motions from performing the same work activities are increased when awkward postures and forceful exertions are involved. Repetitive actions as a risk factor can also depend on the body area and specific act being performed.

### Duration

Job tasks that require use of the same muscles or motions for long durations increase the likelihood of both localized and general fatigue. In general, the longer the period of continuous work the longer the recovery or rest time required.

### Contact Stresses

Repeated or continuous contact with hard or sharp objects, such as nonrounded desk edges or unpadded, narrow tool handles may create pressure over one area of the body (e.g., the forearm or sides of the fingers) that can inhibit nerve function and blood fow.

### Vibration

Exposure to local vibration occurs when a specific part of the body comes in contact with a vibrating object, such as a power hand tool.

### Psychosocial Factors

Identified stressors include the psychological demands of doing meticulous surgery with little or no rest or diversion and time pressures. Dentists with work-related MSDs show a significant tendency to be more dissatisfied at work and to be more burdened by anxiety, experiencing poorer psychoso­matic health and feeling less confident.

## INTERVENTION

Following interventions should be considered in the dental practice:

### Workstation

Proper workstations may include the following:

- Dentist's or patient's chair height
- Lumbar, thoracic or arm support in dentist's chair
- Position of instrument table
- Adequate lighting
- Edges of work surfaces should be comfortable
- Proper ventilation
- Pleasant temperature.^4^

### Early Treatment of MSDs

Early symptoms in the wrist and hand respond to conservative medical management that includes rest, icing, nonsteroidal anti-infammatory drugs and splints. Early intervention could be important in order to achieve a better result at less cost and inconvenience.^5^

### Posture^6^

- Always try to maintain an erect posture
- Use an adjustable chair with lumbar, thoracic and arm support
- Work close to your body
- Minimize excessive wrist movements
- Avoid excessive finger movements
- Alternate work positions between sitting, standing and side of patient
- Adjust the height of your chair and the patient's chair to a comfortable level
- Consider horizontal patient positioning
- Check the placement of the adjustable light.

### Patient Positioning

Supine positioning of the patient in the chair is usually the most effective way to help to maintain neutral posture. The chair should be raised so the operator's thighs can freely turn beneath the patient's chair. Clearance around the patient's head should at least allow unimpeded operator access from the 7 to 12:30 o’clock position, for right-handed operators.

For most intraoral access sites, the maxillary plane should be extended 7° beyond the vertical. For treating the maxillary second and third molars, the maxillary plane should be 25° beyond the vertical. For the mandibular anterior teeth, bring the patients chin down so the maxillary plane is 8°ahead of the vertical.

### Hand Instruments

No industry standard for an ‘ergonomic’ instrument currently exists. A round handle, as opposed to a hexagonal handle, with hard edges will reduce muscular stress and digital nerve compression. However, a smooth, round-handled instrument requires more pinching force to keep the handle from spinning in the hand. Handles with shallow, circumferential grooves or with knurling allow better friction with the fingers so that a secure grasp requires less force. Small diameter, hexagonal shaped instrument handles produce a mechanical stress that may cause digital nerve compression.^7^

When working edges are sharp, the instrument performs more of the work; when the edges are dulled, additional operator force is required to achieve the same result. Sharp instruments are important for reducing excessive force during instrumentation.

### Automatic Instruments

Practitioners should consider use of automatic instruments (high-speed handpiece, slow-speed handpiece, belt driven drills, lasers, ultrasonic scalers, endodontic handpieces) instead of manual hand instruments.

Handpieces should be as light as possible and well balanced. Hose length should be as short as possible; extra hose length adds weight. Avoid retractable or coiled hoses. The tension in the hose is transferred to the wrist and arm as the hose is stretched. Ideally, a pliable hose with a swivel mechanism in the barrel of the hand-piece so that it can rotate with minimal effort should be used.

### Delivery Systems

Various delivery systems have advantages and disadvantages. When working in four-handed dentistry the dentist maintains a position around the operating field with limited hand, arm and body movement, and should best confine eye focus to the working field.

Additionally, the dental equipment and instruments should be centered on the dental assistant. From an ergonomic viewpoint, over-the-head and over-the-patient delivery systems better allow the dental assistant to access the hand-pieces for bur changes or other operations.

### Lighting and Magnification

The goal of overhead lighting is to produce even, shadow-free, color-corrected illumination that is concentrated on the operating field. In general, the intensity ratio between task lighting (the dental operating light) and ambient room lighting should be no greater than 3 to 1.6. Furthermore, the light source should be in the patient's mid-sagittal plane; directly above and slightly behind the patient's oral cavity, and 5° toward the head of the operator in the 12 o’clock position.

Once the patient and operator are properly positioned, the light source can be left far above the heads of both the operator and assistant because the correct position will require no adjustment during the procedure.

### Gloves

Each dental healthcare worker must have gloves of proper size and ft. Although the Influence of gloves on hand discomfort has yet to be explored, they have been cited indirectly as a potential contributor to carpal tunnel syndrome.

### Supervised Exercise

While there is evidence in the literature that poor physical conditioning may increase the risk of musculoskeletal in­jury, there is no empirical support for the success of using stretching or exercise techniques in the prevention of MSDs. Exercise and stretching for the treatment of an MSD should be under the supervision of a physician or physical therapist. Injury could incur or a previous injury might be exacerbated by improperly performed exercises.

### Proper Temperatures

Within the work environment, low room temperatures, manipulation of cold materials or instruments and exposure to cold air exhaust can contribute to low finger temperature. There are no standards for finger temperatures, but it is recommended that hands and fingers be kept above 25° C or 77° F to avoid detrimental effects on dexterity and grip strength.

### Procedures and Administration

The appointment schedule can be used to reduce stress and strain. Alternate easy with difficult cases throughout the day and provide buffer periods that accommodate emergency patients or extra time for difficult procedures or patients.^8^With difficult patients and procedures, alter the sequence of the tasks to be performed, whenever possible. For example, in order to increase task rotation, instead of scaling the entire mouth, then polishing all the teeth followed by fossing, consider doing these tasks a quadrant at a time.

## CONCLUSION

A dentist can spend up to 60,000 hours in a lifetime working in tense and distorted positions, with consequent musculoskeletal problems. Dentistry does not lend itself to good posture; however, it is possible with instruction and practice to correct the harmful postural habits that may be the cause of such stress and pain.

Dental professionals are prone to unique muscle imbalances and require special exercise and ergonomic interventions to maintain optimal health during the course of their career. It is important to not only know what are effective interventions, but also in what sequence to implement them.

Begin to make some changes in the way you practice by incorporating some of these suggestions into your regular routine during the work day. You will find that you have less fatigue at the end of the day, you will experience less pain, and you will be able to provide the quality of service that you and your patients demand.

Here are six keys to wellness to help a dentist to work more comfortably, with less fatigue and extend their career:^7^

1. First and foremost, correct the ergonomic problems in the operatory.
2. Physical therapists, neuromuscular therapist should be consulted for musculoskeletal disorders.
3. Major trigger points should be resolved before any strengthening exercise is attempted.
4. Strengthen specific stabilizing muscles (like shoulder and back).
5. Be patient, but most of all commit to a regular regimen of prevention strategies.
6. Chairside stretching is an important strategy to perform throughout the workday to prevent microtrauma and muscle imbalances.
